# Incarcerated inguinoscrotal bladder herniation: A case report highlighting large bladder herniation with intrasaccular calculi

**DOI:** 10.1016/j.eucr.2026.103622

**Published:** 2026-09-23

**Authors:** Josiah Dallmer, Nasser Alabdulaly, Gerard Lin, Leo Zhao, Gavin Scott, Michael J. Whalen

**Affiliations:** a The George Washington University School of Medicine and Health Sciences, 2300 I Street NW, Washington, DC, 20037, USA; b Philadelphia College of Osteopathic Medicine, 625 Old Peachtree Road, Suwanee, GA, USA; c Department of Urology, The George Washington University School of Medicine and Health Sciences, 2150 Pennsylvania Ave NW, Washington, DC, 20037, USA

**Keywords:** Bladder calculi, Bladder herniation, Hernia repair, Urinary retention, Inguinoscrotal herniation

## Abstract

Inguinal herniation of the bladder is a rare urologic occurrence, with intrascrotal bladder herniation containing calculi an exceptionally uncommon presentation. We report a 79-year-old African American man with chronic urinary retention and prior irradiation for prostate cancer who presented with a right inguinal hernia containing the majority of the bladder with intraluminal calculi. Intraoperatively, the bladder was found to be incarcerated despite a preoperative description as reducible. Despite proper anatomic restoration of the bladder, the patient failed a voiding trial at 2 months and suffered 3 hospitalizations over the follow-up period, highlighting the longer-term urologic sequelae of chronic bladder dysfunction.

## 1 Introduction

Involvement of the urinary bladder in inguinal hernia cases is uncommon, occurring in an estimated 1–4% of cases and predominantly in obese men beyond the fifth decade of life with chronic bladder outlet obstruction (BOO) or urinary retention.^1^^,^^2^ The condition is rarely recognized before operation due to the absence of cross-sectional imaging before routine inguinal hernia repairs, and fewer than 7% of cases are diagnosed preoperatively. The majority of cases are identified intraoperatively or postoperatively, often following inadvertent bladder injury.^3^^,^^4^ Affected patients may be asymptomatic or report obstructive urinary symptoms such as a weak stream, urinary intermittency, incomplete emptying, and straining with urination. The diagnosis is frequently complicated by recurrent urinary tract infection or BOO.^5^ Given the diagnostic difficulty, cross-sectional imaging is essential to characterize the contents of the hernia sac before repair and to avoid iatrogenic injury.^2^^,^^3^ Rarer still is the formation of calculi within the herniated bladder segment, attributed to urinary stasis within the dependent pouch and described almost exclusively in isolated case reports.^6^^,^^7^

This case highlights the interplay of urologic comorbidities predisposing to inguinoscrotal bladder herniation with intrasaccular calculi and contributes to the limited literature characterizing this rare clinical presentation.

## 2 Case presentation

We report the case of a 79 year old African American male with a significant urologic history who presented to the emergency department with a UTI in the setting of a known right bladder inguinal herniation into the scrotum. His medical history was notable for diabetes mellitus, high-risk, locally advanced prostate adenocarcinoma (cT1c, cN1, cM0, PSA 360 ng/mL, Grade Group 5) managed with androgen deprivation therapy (ADT) followed by one month of external radiation beam therapy (XRT) approximately 14 months before surgical repair of bladder herniation, benign prostatic hyperplasia (BPH) complicated by cystolithiasis and recurrent UTI, and chronic urinary retention managed with a pre-existing suprapubic catheter. Videourodynamic studies (VUDS) demonstrated a weak but present detrusor contraction with intact sensation and an inability to void, with a bladder compliance of 24.4 mL/cmH_2_O, consistent with reduced compliance in the setting of chronic bladder outlet obstruction. Years ago, he underwent transurethral resection of the prostate (TURP) for his BPH despite a prostate volume of 157 cc on magnetic resonance imaging (MRI), taking this approach instead of guideline-concordant simple prostatectomy or HoLEP as the patient did not want extirpative treatment.

The patient initially presented to the ED with abdominal pain, UTI, and a right inguinal bulge that had been identified on imaging as a bladder herniation 8 months prior at an outside institution, prompting further evaluation with computed tomography of the abdomen and pelvis (CTAP) with contrast. This demonstrated a right inguinal hernia with a neck measuring 3.4cm, with the hernia sac containing the majority of the bladder (Fig. 1). Within the herniation, multiple calculi were identified measuring up to 1cm along with foci of air, with additional scattered calculi identified within the non-herniated portion of the bladder lumen. The bladder wall was circumferentially thickened, consistent with chronic cystitis. Additional CTAP findings included multiple nonobstructing bilateral renal calculi with the largest being 1.1 × 0.7cm in the left kidney, and an 8mm left ureterovesical junction calculus with trace distal hydroureter and no hydronephrosis. Upon physical examination, the hernia was initially described as reducible with no tenderness to palpation; however, staff did note severe lower abdominal tenderness with guarding. Vitals on admission were a temperature of 37C, 144/72 BP, and 95 HR. Laboratory evaluation at the time of admission revealed leukocytosis (WBC 12.67 × 10^3^/μL), creatinine 1.4 mg/dL, blood urea nitrogen 24 mg/dL, and a serum glucose of 182 mg/dL. Urinalysis demonstrated many bacteria, positive leukocyte esterase, negative nitrites, 538 leukocytes/HPF, hemoglobin, urine glucose greater than 1000mg/dL, and trace ketones with hyaline casts. Urine culture grew *Klebsiella oxytoca* and *Raoultella ornithinolytica* at greater than 100,000 CFU/mL, and the patient was treated with empiric antibiotic therapy that was narrowed when urine culture results returned.

**Fig. 1:**
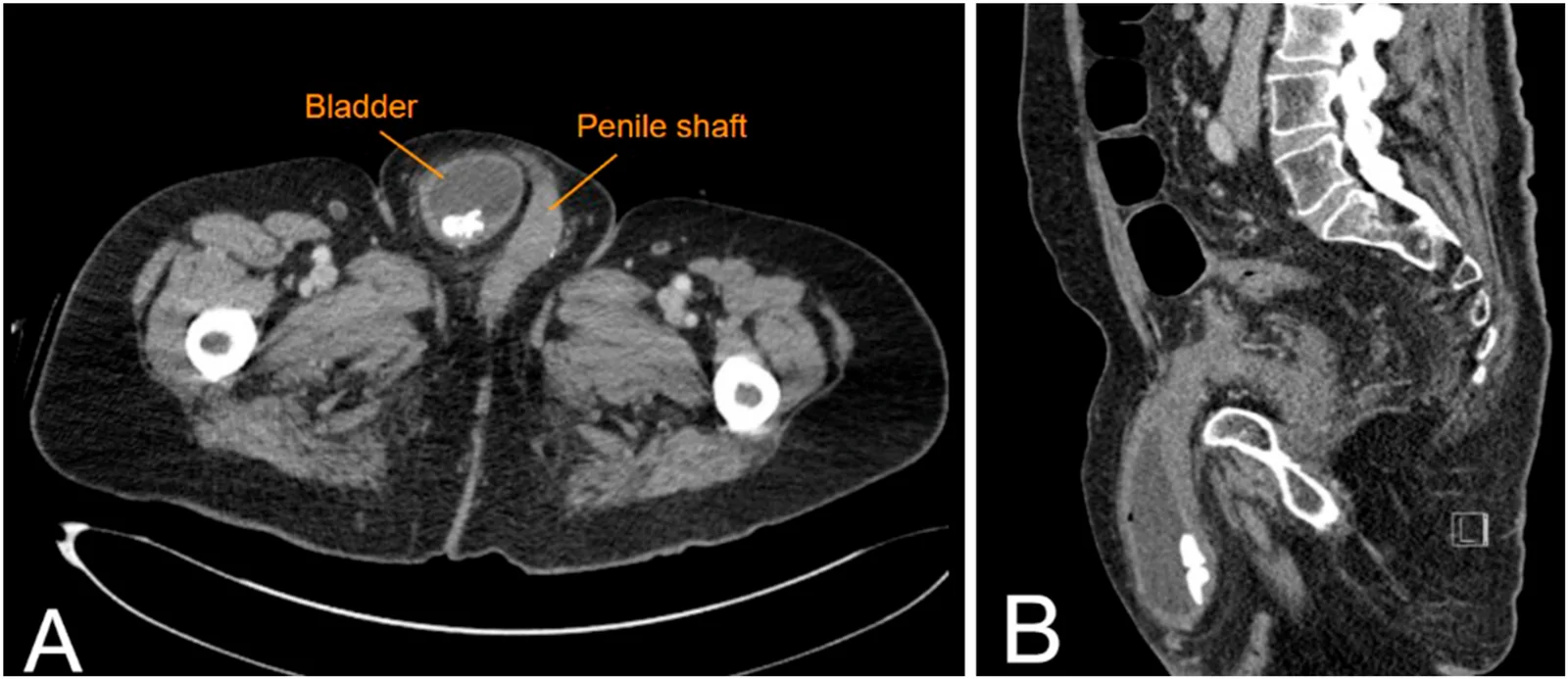
Computed tomography (CT) imaging A. CT abdomen/pelvis demonstrating bladder herniation into the scrotum and bladder calculi - axial view B. View demonstrating the extent of bladder herniation - sagittal view.

The patient had been scheduled for elective hernia repair in coordination with urology; however, following admission for UTI, general surgery proceeded with open inguinal hernia repair during this hospitalization. An inguinal incision was made and dissection was carried down to the level of the hernia sac, where it was found that the hernia had both an indirect and direct component, and that the bladder was incarcerated in contrast to its preoperative characterization as reducible. The bladder was carefully mobilized to the internal inguinal ring. Following confirmation of hemostasis, it was then further mobilized back into the pelvis. The inguinal floor was reinforced using keyhole polypropylene mesh. Later that day, urology performed a bedside exchange of the pre-existing suprapubic catheter with a 16-French latex suprapubic catheter.

Pathologic examination of the resected hernia sac contents revealed nothing of note. The patient had an uncomplicated postoperative stay and was discharged after 3 days. Cystolitholopaxy was not performed at time of hernia repair secondary to active UTI. The patient was discharged with trimethoprim-sulfamethoxazole for continuing treatment of his UTI, and was later prescribed alfuzosin at his one-week outpatient urology follow-up with the expectation that restoration of normal bladder anatomy might facilitate voiding recovery.

He has had no hernia recurrence as of his latest follow-up 10-months post-operatively. At his 2-month postoperative outpatient visit, the patient failed a voiding trial, resulting in the maintenance of the suprapubic catheter and he has unfortunately also had multiple UTIs requiring hospitalization.

## 3 Discussion

Bladder involvement in inguinal hernias is relatively uncommon, encompassing an estimated 1-3% of inguinal hernias with a higher proportion in men over 50 years of age.^8^ Recognized predisposing factors include male sex, advanced age, obesity, bladder outlet obstruction, chronic bladder distention, decreased bladder tone, and weakening of the abdominal wall and pelvic supporting structures. Clinical presentation depends on the extent of herniation: small bladder hernias are typically asymptomatic, while larger bladder hernias classically present with two-stage micturition.^9^

Prior case reports discuss that bladder calculi associated with inguinoscrotal bladder herniation likely arise from urinary stasis within the herniated bladder segment.^7^^,^^10^ Two stage micturition commonly observed in inguinoscrotal bladder herniation implies that the herniated portion of the bladder acts as a reservoir for urine that incompletely empties during voiding, permitting crystals to aggregate around a nidus and develop into calculi. This mechanism is particularly relevant when bladder diverticula are involved, as the absence of detrusor muscle within the diverticular wall prevents contraction. In our patient, a stone was identified within a left posterior bladder diverticulum on imaging, further illustrating how areas of impaired drainage increase the risk of calculus formation. Intraoperative identification that the bladder was incarcerated further supports that the herniated component had impaired drainage. In our patient, several additional features likely contributed to secondary stone formation. The patient's chronic BOO, recurrent UTIs, and chronic catheterization are recognized risk factors for the formation of secondary bladder stones. The elevation in intravesical pressure from chronic BOO may have also predisposed the bladder to herniation through an inguinal defect. Finally, the presence of bilateral renal calculi and a ureterovesical junction calculus suggests an additional component of urinary supersaturation driving upper tract stone formation, distinct from the stasis-mediated mechanism underlying bladder and intrasaccular calculi.

Although the hernia was initially described as reducible on physical examination, intraoperative exploration demonstrated an incarcerated hernia. This discrepancy highlights an important diagnostic challenge in inguinoscrotal bladder herniation. Unlike bowel-containing inguinal hernias, which typically present with signs of gastrointestinal obstruction such as nausea, vomiting, or obstipation, bladder hernias may present more insidiously. In this case, the patient's pre-existing suprapubic catheter likely led to continuous partial decompression of the herniated bladder, making the hernia appear smaller and more compressible on palpation. In patients without a suprapubic catheter, fluctuations in scrotal size would typically raise clinical suspicion for bladder involvement.^11^ Because missed bladder involvement increases the risk of iatrogenic injury during hernia repair, accurate preoperative diagnosis is important for operative planning and prevention of bladder injury. In our patient, CT not only confirmed the extent of bladder herniation and the presence of intrasaccular calculi but also identified additional pathology including a ureteral and bilateral renal calculi. Therefore, a CT scan should be strongly considered in older men with inguinoscrotal hernias and urinary symptoms or complex urologic history.

This patient's complex urologic history, including high-risk locally advanced prostate cancer managed with XRT and ADT, recurrent UTIs, and chronic urinary retention, contributes to his morbidity. ADT is effective in reducing prostate volume and tumor burden, however, it has a complex effect on bladder function. It can improve LUTS secondary to outlet obstruction by reducing prostate volume by approximately 43% at 6 months of treatment.^12^ However, research shows that ADT may simultaneously impair bladder function through direct effects on the detrusor. In vivo experimental studies have demonstrated that ADT induces bladder remodeling through smooth muscle atrophy, reduced smooth muscle to collagen ratio, and increased fibrosis.^13^ Clinically, a population-based cohort study found that ADT was associated with a 1.21-fold increased risk of acute urinary retention.^14^ In our patient, who had pre-existing detrusor dysfunction on urodynamics, ADT-induced bladder remodeling may have further compromised an already decompensated detrusor, perpetuating chronic urinary retention and the resultant urinary stasis that promotes calculus formation.

Furthermore, radiation therapy introduces additional considerations that increase herniation susceptibility. A large population-based Swedish study demonstrated that men who received radiation therapy for prostate cancer had an almost two-fold increased incidence of groin hernia repair compared with age-matched controls.^15^ This is due to the fact that pelvic radiation induces chronic inflammation, fibrosis, and impaired collagen remodeling in irradiated tissues.^16^ These changes may compromise tensile strength of the inguinal floor and predispose patients to hernia formation. Notably, the sequence of TURP followed by pelvic radiation in this patient carries a particular risk, as radiation to a post-TURP area may compound sphincter injury and promote bladder neck contracture. Our patient's failure to recover voiding function after his hernia repair, evidenced by a failed voiding trial at two months postoperatively and continued dependence on suprapubic catheter drainage, reflects the cumulative impact of these insults on detrusor function.

Regarding surgical approach, no consensus exists for the cystolithiasis subset specifically. Some cases document the open Lichtenstein approach, while others have described a laparoscopic transabdominal preperitoneal approach with a modified peritoneal incision as an alternative, and at least one report removed calculi in a single stage directly through the hernia incision.^10^^,^^17^^,^^18^ In the case presented here, stone management was deferred due to the active UTI at the time of surgical repair, consistent with the HerniaSurge systematic review recommendations of perioperative antibiotic prophylaxis and urinary catheterization for complex presentations.^4^

Postoperative voiding outcomes after bladder herniations in this manner are also documented rarely. Celik et al. described a patient with complete bladder herniation and acute renal failure who achieved spontaneous voiding after three months of intermittent self-catheterization and daily alpha-blocker use following reduction, while Yustira et al. utilized serial uroflowmetry to objectively document satisfactory voiding outcomes after herniorrhaphy requiring partial cystectomy.^19^^,^^20^ Critically, neither report describes preoperative bladder function, limiting meaningful comparison to our patient. His failure to void despite anatomic restoration of the bladder suggests that underlying detrusor dysfunction may limit voiding recovery regardless of successful hernia reduction. It therefore remains unclear whether restoring correct anatomic bladder position confers meaningful voiding benefit in patients with pre-existing detrusor weakness or outlet obstruction.

## 4 Conclusion

This case highlights a rare presentation of intrascrotal bladder herniation with intrasaccular calculi. While individual predisposing factors such as chronic urinary retention, bladder outlet obstruction, and recurrent urinary tract infections are well-documented contributors to bladder herniation and secondary stone formation, this patient's history of pelvic irradiation for prostate cancer and prior TURP likely compounded his susceptibility through progressive weakening of pelvic floor and inguinal supporting structures. Furthermore, the discrepancy between preoperative characterization as reducible and intraoperative characterization as incarcerated highlights a diagnostic challenge inherent to these cases. Continued reporting of such cases is essential to better characterize this subset of hernias and improve long-term outcomes for this complex patient population.

## CRediT authorship contribution statement

**Josiah Dallmer:** Conceptualization, Data curation, Methodology, Project administration, Investigation, Writing – original draft, Writing – review & editing. **Nasser Alabdulaly:** Validation, Writing – original draft, Writing – review & editing. **Gerard Lin:** Methodology, Writing – original draft. **Leo Zhao:** Validation, Writing – original draft. **Gavin Scott:** Writing – original draft. **Michael J. Whalen:** Conceptualization, Methodology, Project administration, Supervision, Writing – original draft, Writing – review & editing.
